# SARS-CoV-2 infects human neural progenitor cells and brain organoids

**DOI:** 10.1038/s41422-020-0390-x

**Published:** 2020-08-04

**Authors:** Bao-Zhong Zhang, Hin Chu, Shuo Han, Huiping Shuai, Jian Deng, Ye-fan Hu, Hua-rui Gong, Andrew Chak-Yiu Lee, Zijiao Zou, Thomas Yau, Wutian Wu, Ivan Fan-Ngai Hung, Jasper Fuk-Woo Chan, Kwok-Yung Yuen, Jian-Dong Huang

**Affiliations:** 1 School of Biomedical Sciences, Li Ka Shing Faculty of Medicine, The University of Hong Kong, 3/F, Laboratory Block, 21 Sassoon Road, Pokfulam, Hong Kong China; 2 CAS Key Laboratory of Quantitative Engineering Biology, Shenzhen Institute of Synthetic Biology, Shenzhen Institutes of Advanced Technology, Chinese Academy of Sciences, Shenzhen, Guangdong 518055 China; 3 Department of Microbiology, Li Ka Shing Faculty of Medicine, The University of Hong Kong, Pokfulam, Hong Kong China; 4 Department of Medicine, Li Ka Shing Faculty of Medicine, The University of Hong Kong, Pokfulam, Hong Kong China; 5 Guangdong-Hongkong-Macau Institute of CNS Regeneration (GHMICR), Jinan University, Guangzhou, Guangdong 510632 China; 6 Re-Stem Biotech, Suzhou, Jiangsu 330520 China; 7 State Key Laboratory of Emerging Infectious Diseases, The University of Hong Kong, Pokfulam, Hong Kong China

**Keywords:** Neural stem cells, Stem cells, Mechanisms of disease

Dear Editor,

Coronavirus disease 2019 (COVID-19) caused by the novel severe acute respiratory syndrome coronavirus 2 (SARS-CoV-2)^1^ has resulted in over 13 million confirmed cases and more than 580,045 deaths across 218 countries and geographical regions as of July 16, 2020. This novel coronavirus primarily causes respiratory illness with clinical manifestations largely resembling those of SARS. However, neurological symptoms including headache, anosmia, ageusia, confusion, seizure, and encephalopathy have also been frequently reported in COVID-19 patients.^2,3^ In a study of 214 hospitalized COVID-19 patients in Wuhan, China, neurologic findings were reported in 36.4% of patients, and were more commonly observed in patients with severe infections (45.5%).^2^ Similarly, a study from France reported neurologic findings in 84.5% (49/58) of COVID-19 patients admitted to hospital.^3^ Importantly, a recent study in Germany demonstrated that SARS-CoV-2 RNA could be detected in brain biopsies in 36.4% (8/22) of fatal COVID-19 cases,^4^ which highlights the potential for viral infections in the human brain. To date, there has been no direct experimental evidence of SARS-CoV-2 infection in the human central nervous system (CNS). We recently demonstrated that SARS-CoV-2 could infect and replicate in cells of neuronal origin.^5^ In line with this finding, we showed that SARS-CoV-2 could infect and damage the olfactory sensory neurons of hamsters.^6^ In addition, angiotensin-converting enzyme 2 (ACE2), the entry receptor of SARS-CoV-2, is widely detected in the brain and is highly concentrated in a number of locations including substantia nigra, middle temporal gyrus, and posterior cingulate cortex.^7^ Together, these findings suggest that the human brain might be an extra-pulmonary target of SARS-CoV-2 infection.

To explore the direct involvement of SARS-CoV-2 in the CNS in physiologically relevant models, we assessed SARS-CoV-2 infection in induced pluripotent stem cells (iPSCs)-derived human neural progenitor cells (hNPCs), neurospheres, and brain organoids.^8^ We first evaluated the expression of ACE2 and key coronavirus entry-associated proteases in hNPCs. Our data suggested that ACE2, TMPRSS2, cathepsin L, and furin were readily detected in the hNPCs (Supplementary information, Fig. S1). Next, we challenged iPSC-derived hNPCs with SARS-CoV-2 at 10 multiplicity of infection (MOI) and with SARS-CoV as a control. Supernatant was harvested at 0, 24, and 48 h post infection (hpi) for virus replication assessment. Interestingly, our data suggested that SARS-CoV-2, but not SARS-CoV, could replicate in hNPCs (Fig. 1a; Supplementary information, Fig. S2). In addition, we quantified the cell viability of SARS-CoV-2-infected hNPCs. Importantly, SARS-CoV-2 infection significantly reduced the viability of hNPCs to 4.7% (*P* < 0.0001) and 2.5% (*P* < 0.0001) of that of the mock-infected hNPCs at 72 and 120 hpi, respectively (Fig. 1a). In contrast to the substantial cytotoxicity induced by SARS-CoV-2 in the infected hNPCs, SARS-CoV-2 infection did not significantly upregulate interferon (Supplementary information, Fig. S3) and pro-inflammatory (Supplementary information, Fig. S4) response in the infected hNPCs. Next, we challenged 3D neurospheres with SARS-CoV-2 and harvested supernatant samples from the infected neurospheres at 0, 24, 48, and 72 hpi for virus replication assessment. We found the SARS-CoV-2 RNA-dependent RNA polymerase (RdRp) copy number significantly increased in a time-dependent manner (Fig. 1b, left). In addition, a significant amount of infectious virus particles were released from the infected neurospheres as determined by plaque assays (Fig. 1b, right). In parallel, SARS-CoV-2-infected neurospheres were cryosectioned and immunostained for viral antigen assessment. Importantly, SARS-CoV-2 nucleocapsid (N) protein was readily detected across the infected neurospheres but no positive signals were detected in the mock-infected neurospheres (Fig. 1c). Furthermore, electron microscopy detected extensive viral particles in vacuoles within the double-membrane structures, which may represent sites of viral particle formation (Fig. 1d). These findings indicate that neurospheres were permissive to SARS-CoV-2 infection and supported productive virus replication.

**Fig. 1:**
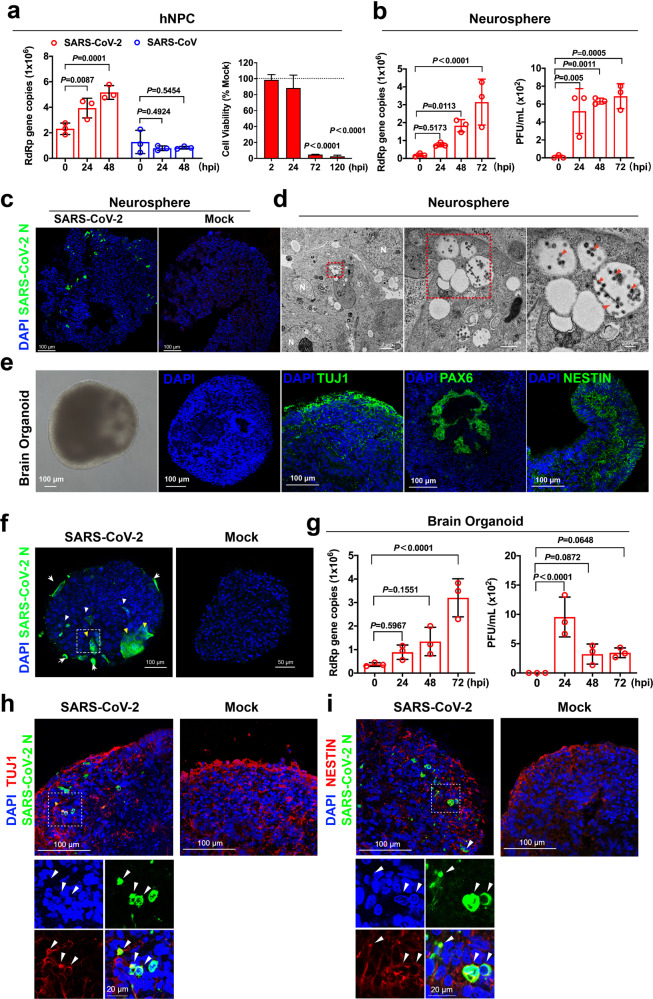
SARS-CoV-2 infects hNPCs, neurospheres and brain organoids. **a** hNPCs were challenged with 10 MOI SARS-CoV-2 or SARS-CoV. Viral supernatant samples were harvested at 0, 24, and 48 hpi and viral loads were determined by qRT-PCR. The cell viability of SARS-CoV-2 and mock-infected hNPCs were quantified with CellTiterGlo assays. Bars represent means ± SD of three independent experiments. **b** Supernatant samples from SARS-CoV-2-inoculated neurospheres were analyzed for viral RNA by qRT-PCR. Bars represent mean ± SD. Infectious virus titer of the supernatant samples from SARS-CoV-2-inoculated neurospheres were determined by plaque assay. Data were obtained from three (*n* = 3) independent batches of neurospheres in three experiments. **c** Representative confocal images of SARS-CoV-2- or mock-inoculated neurospheres harvested at 72 hpi. SARS-CoV-2 was identified with a SARS-CoV-2 nucleocapsid (N) protein immune serum (green). Scale bars, 100 µm. **d** Representative transmission electron microscopy images of SARS-CoV-2-inoculated neurospheres at 72 hpi. Complete SARS-CoV-2 particles (red arrowheads), and nucleus (white N). Scale bars, 2 µm, 500 nm, or 200 nm. **e** Characterization of 35-day-old brain organoids. Brain organoids were fixed with 4% paraformaldehyde, and then transferred to 30% sucrose solution and embedded for cryosectioning. A representative bright-field image of brain organoid was shown. DAPI-stained 35-day-old brain organoid showing complex inner morphology. Representative images of 35-day-old human brain organoids immunostained for neuronal cell marker TUJ1, radial glial cell marker PAX6, and proliferation neural progenitor cell marker NESTIN. Scale bars, 100 µm. **f** Representative images of SARS-CoV-2-infected brain organoids. SARS-CoV-2 was detected using a SARS-CoV-2-N immune serum (green). SARS-CoV-2 infected cells were identified at the peripheral region (arrows) and in deeper regions (white arrowheads) of the organoids. Substantial cell-cell fusion was detected (yellow arrowheads). No positive N signals were detected in mock-infected brain organoids. Scale bars, 100 µm. **g** Viral supernatant samples were harvested at 0, 24, 48, and 72 hpi. Virus replication was detected by qRT-PCR. Infectious virus titer was determined by plaque assay on Vero E6 cells. Statistical significance was determined by one-way ANOVA. Bars represent the means ± SD of three independent experiments. **h** Representative images of SARS-CoV-2-infected human brain organoids immunostained for SARS-CoV-2-N and TUJ1. Scale bars, 100 µm or 20 µm. **i** Representative images of SARS-CoV-2-infected human brain organoids immunostained for SARS-CoV-2-N and NESTIN. Scale bars, 100 µm or 20 µm. Confocal images were obtained on a Zeiss LSM880 confocal imaging system. The images were representative of three (*n* = 3) independent batches of neurospheres or organoids from three experiments. Statistical significance was determined by two-way ANOVA. MOI multiplicity of infection, hpi hours post inoculation.

Next, we examined whether SARS-CoV-2 could infect 3D human brain organoids. We generated iPSC-derived human brain organoids using previously described protocols.^8^ The 35-day-old brain organoids showed self-organizing internal morphology with fluid-filled ventricular-like structures resembling that of developing cerebral cortex (Fig. 1e). Cryosectioning and immunostaining were performed to determine the expression and distribution of neuronal markers in 35-day-old brain organoids. Pan-neurons, early forebrain, and hNPCs markers were identified by TUJ1, PAX6, and NESTIN staining, respectively. The TUJ1 staining identified a primitive cortical plate with early neurons (Fig. 1e), whereas PAX6 staining represented the radial glia in the cerebral cortex (Fig. 1e). In addition, NESTIN staining identified active proliferating NPCs in the brain organoids (Fig. 1e). Overall, these results indicated that telencephalon development and cerebral neurogenesis could be modelled by our organoid system.

To investigate whether the brain organoids were permissive to SARS-CoV-2 infection, human iPSC-derived 35-day-old brain organoids were challenged with SARS-CoV-2. Importantly, extensive SARS-CoV-2 antigen was detected in the infected samples at 72 hpi (Fig. 1f), indicating that SARS-CoV-2 directly infected the brain organoids. Immunofluorescence staining and confocal microscopy revealed SARS-CoV-2-N signals in the peripheral regions (Fig. 1f, arrows) and in deeper regions of the brain organoids (Fig. 1f, white arrowheads). In addition, cell-cell fusion was readily detected in regions with robust SARS-CoV-2 infection (Fig. 1f, yellow arrowheads). No SARS-CoV-2-N signals were detected in the mock-infected brain organoids (Fig. 1f). We next analyzed supernatant samples from infected brain organoids to evaluate SARS-CoV-2 virus particle release. The results demonstrated SARS-CoV-2 RdRp gene copy number increased in a time-dependent manner, suggesting active release of progeny virus particles from infected brain organoids (Fig. 1g, left). Specifically, about 3.2 × 10^6^ copies of SARS-CoV-2 RdRp gene were detected at 72 hpi, which was nine-fold higher than at 0 hpi (*P* < 0.0001) (Fig. 1g, left). Plaque assays performed on supernatant samples from brain organoids infected with SARS-CoV-2 showed that the infectious virus titer peaked at 24 hpi and were continuously detected at 48 and 72 hpi. These findings unambiguously demonstrated that SARS-CoV-2 can productively infect brain organoids with release of viral particles (Fig. 1g, right). Remarkably, double immunostaining demonstrated that SARS-CoV-2-N was colocalized with neuronal marker TUJ1 and NPC marker NESTIN, suggesting that SARS-CoV-2 can directly infect cortical neurons and NPCs in brain organoids (Fig. 1h, i).

In summary, our results demonstrated that iPSC-derived hNPCs were permissive to SARS-CoV-2, but not SARS-CoV infection. Extensive viral protein expression and infectious viral particles were detected in neurospheres and brain organoids infected with SARS-CoV-2, which suggest SARS-CoV-2 can productively infect the human brain. Importantly, SARS-CoV-2 infection in 3D human brain organoids was localized to TUJ1- and NESTIN-positive cells, suggesting SARS-CoV-2 could directly target cortical neurons and NPCs. The neurosphere model represents early characteristics of neurogenesis, whereas the brain organoid model exhibits features of human cortical development that recapitulate the development and physiological arrangements of the human brain.^9^ The finding that SARS-CoV-2 can productively infect human brain organoids highlights the potential of direct viral involvement in neurological symptoms in COVID-19 patients. These results provided insight on the pathognomonic symptoms of anosmia (loss of smell) and ageusia (loss of taste) as well as other neurological manifestations of COVID-19 including seizure, encephalopathy, encephalitis, Guillain-Barre syndrome, and Miller Fisher syndrome. During the submission process of this manuscript, the susceptibility of human brain organoid was similarly suggested by two independent studies.^10,11^ However, the permissiveness of neuronal progenitor cells to SARS-CoV-2 was not evaluated in these studies.^10,11^ We demonstrated here that SARS-CoV-2 could also target the neuronal progenitor cell populations. In this regard, the recovery of the olfactory function and other neurological manifestations might be incomplete and late as these neural progenitor cells could be infected by SARS-CoV-2. Importantly, the Zika virus that re-emerged in 2015 is also well recognized to target neural progenitor cells in the human brain, leading to microcephaly and severe developmental defects in the fetus and other neurological anomalies in adults.^12^ The chronic or long-term consequence of SARS-CoV-2 infection in the CNS should be closely monitored.

## Supplementary information

Supplementary Information
