# Supplementary material for: SARS-CoV-2 infects human neural progenitor cells and brain organoids
Source: Cell Res. 2020 Aug 4;30(10):928–31. doi: 10.1038/s41422-020-0390-x (PMC7399356; doi:10.1038/s41422-020-0390-x)
Supplement: Supplementary file 1 — Supplementary Information [file 41422_2020_390_MOESM1_ESM.pdf]

# Supplementary Materials

# SARS-CoV-2 infects human neural progenitor cells and brain organoids

## Materials and Methods

### Human neural progenitor cells

hNPCs were generated from pluripotent stem cells (iPSCs) as we previously described [1]. In brief, iPSCs were maintained in mTeSRTM1 medium (STEMCELL Technologies, Vancouver, Canada) and were induced for differentiation into hNPCs using a basal medium with a cocktail of supplements including 1 μM A83-01 (SelleckChem), 5 μM CHIR99021 (SelleckChem), 0.3 μM LDN-193189 (MedChemExpress), 0.1 μM of all trans retinoic acid (Sigma), 1 μM RO4929097 (SelleckChem), and 1 μM SU-5402 (SelleckChem). Every 25 mL of basal medium was prepared by 12.5 mL of advance DMEM/ F12 medium (Gibco) and 12.5 mL of neurobasal medium (Gibco) with 10 μg/mL human insulin (Sigma), 250 μL 50× B27 supplement (Gibco), 125 μL 100× N2 supplement (Gibco), 125 μL 100× GlutaMAX (Gibco), 10 μM β- mercaptoethanol (Gibco), and 250 μL 100× P/S. The iPSCs were differentiated for a total of 9 days, during which the media was replaced every two days during the first 4 days and refreshed every day in the following 5 days. The differentiated hNPCs were then seeded in Matrigel-coated plate for infection.

### Formation of neurospheres

Neurospheres were generated as previously described [2]. Briefly, iPSCs were separated using Accutase (Thermo Fisher Scientific) and centrifuged, and then resuspended in medium containing 15% fetal bovine serum, IMDM medium, 2 mM L-glutamine, 100 U PS, 1% NEAA, 10 mM β-mercaptoethanol, 1 mM sodium pyruvate, 200 mg/mL iron-saturated transferrin, and 50 mg/mL ascorbic acid supplemented with 1 mM Dorsomorphin (STEMCELL Technologies) and 10 mM SB (Stemgent, SB431542). Cells were plated on low attachment 96-well plates to form embryoid body (EB). After 7 days, the EBs were plated to form rosettes expressing neural progenitors using

defined medium DMEM/F-12 supplemented with 20 ng/mL FGF2 and Gem21 NeuroPlex (Gemini Bio-Products). For neurosphere generation, 4,000 neural progenitor cells were seeded on low attachment plates under rotation without FGF2.

**Formation of brain organoids**

Human brain organoids were generated from iPSCs as previously described [2,3]. Briefly, feeder-free iPSCs were fed with mTeSR1 for 7 days. Colonies were dissociated in PBS (1:1) with Accutase for 10 minutes, and then centrifuged at 150 x *g* at 37°C for 3 minutes. The pellet was resuspended in mTeSR1 supplemented with 10 mM SB and 1 mM Dorso. Approximately 5 x $10^6$ cells were transferred to one well in a 6-well plate in presence of 5 μM ROCK inhibitor (STEMCELL Technologies) and kept under suspension by rotation (95 rpm) for 24 hours to form free-floating spheres. After 3 days, mTeSR1 was replaced by neurobasal Media I containing GlutaMAX 1 (Thermo Fisher Scientific), 1% NEAA (Thermo Fisher Scientific), 1% Gem21 NeuroPlex, 1% N2 NeuroPlex (Gemini Bio-Products), 10 μM SB, 1 mM Dorso, and 1% PS (Life Technologies), and cells were maintained for 1 week. The media was changed to neurobasal Media II containing GlutaMAX 1, 1% NEAA, 1% Gem21 NeuroPlex, and 1% PS supplemented with 20 ng/mL FGF2 (Thermo Fisher Scientific), and cells were maintained for another week. Cells were transferred to Media III containing 10 ng/mL GDNF, 10 ng/mL BDNF, 10 ng/mL NT-3 (PeproTech), 1 mM dibutyryl-cAMP (Sigma-Aldrich), and 200 mM L-ascorbic acid. After a further week in Media III, the organoids were transferred to Media II and incubated until day 35 with the media refreshed every 3 days.

**Viruses and biosafety**

The SARS-CoV-2 HKU-001a (GenBank accession number: MT230904) and SARS-CoV GZ50 (GenBank accession number: AY304495) were propagated as previously described [4]. Both viruses were titered in Vero E6 cells by plaque assay. All experiments involving live SARS-CoV-2 and SARS-CoV followed the approved standard operating procedures in our Biosafety Level 3 facility [4].

**Infection of neurospheres or organoids with SARS-CoV-2**

To evaluate whether neurospheres or organoids were permissive to SARS-CoV-2, neurospheres or brain organoids were inoculated with $6 \times 10^6$ PFU/mL SARS-CoV-2 in organoid culture medium and incubated at 37°C for 24 hours. The inoculum was aspirated at 24 hours post virus challenge. Organoids were washed with culture medium three times and then further incubated until harvest at the indicated time points.

**Infection of hNPCs with SARS-CoV-2 or SARS-CoV**

To evaluate whether hNPCs were permissive to SARS-CoV-2 infection, differentiated hNPCs were challenged with 10 MOI SARS-CoV-2 or SARS-CoV. Supernatant samples from cells were harvested at 0, 24, and 48 hpi. Samples were lyzed with AVL buffer (Qiagen) and virus replication was determined by qRT-PCR.

**Immunostaining and confocal microscopy**

Immunofluorescence staining and confocal microscopy were performed as previously described [5] with slight modification. Briefly, organoids were fixed in fresh paraformaldehyde (4% PFA, Sigma-Aldrich) at pH 7.4 and 4°C overnight. Organoids were then transferred to 30% (wt/vol) sucrose solution in PBS at 4°C overnight. Organoids were embedded in embedding medium containing 10% (wt/vol) sucrose and 7.5% (wt/vol) gelatin in PBS. Organoids were cryosectioned at a thickness of 16 μm using a CryoStar NX50 Cryostat (Thermo Fisher Scientific). After washing in PBS buffer, organoids were permeabilized in 0.5% PBST (0.5% Triton-X in PBS buffer) for 3 hours at room temperature. Tissues were blocked with blocking medium containing 3% BSA and 0.025% PBST (0.025% Tween-20 in PBS buffer) for 1 hour. Tissues were stained for neuronal markers using mouse primary anti-beta III Tubulin antibody (1:100) (Abcam, #ab78078), rabbit anti-PAX6 antibody (1:100) (Abcam, #ab5790), goat anti-SOX2 antibody (1:400) (R&D system, #AF2018), and rabbit anti-NESTIN antibody (1:100) (MilliporeSigma, #ABD69). The SARS-CoV-2 nucleocapsid (N) protein was identified using an in-house mouse anti-SARS-CoV-N serum or in-house rabbit anti-SARS-CoV nucleocapsid serum, which cross-reacts with SARS-CoV-2-N as we previously described [5]. Tissue slides were labeled with

primary antibodies diluted in PBS with 2% FBS at 4°C overnight. After washing with 0.025% PBST, secondary antibodies including donkey anti-rabbit IgG Alexa Fluor 568 (1:1000) (Invitrogen, #A-10042), donkey anti-mouse IgG 488 (1:1000) (Invitrogen, #A-21202), and donkey anti-rabbit Alexa Fluor 647 (1:1000) (Invitrogen, #A-31573) together with DAPI (Abcam, #ab228549) were applied to the sections for 2 hours at room temperature. After washing with 0.025% PBST, slides were mounted with ProLong Glass Antifade Mountant (Thermo Fisher Scientific, P36984) and examined by confocal microscopy using a Zeiss LSM 880 system (Zeiss), and images were processed with ImageJ.

**Transmission electron microscopy**

Infected or mock-infected spheres were washed once in PBS and fixed with 2.5% glutaraldehyde at $4^{o}$C overnight. The fixed organoids and neurospheres were post-fixed with 1% osmium. The samples were embedded in epoxy resin and processed into ultrathin sections using an Ultracut UCT Ultramicrotome (Leica). The sections were stained with uranyl acetate and Reynold's lead citrate. Images were obtained on an FEI Tecnai $G^2$ 20 S-TWIN transmission electron microscope (FEI Company).

**Extraction and qRT-PCR for detecting SARS-CoV-2 replication and host gene expression**

Supernatant was harvested at 0, 24, 48, and 72 hpi from organoids, neurospheres, and hNPCs. Viral RNA was extracted using a QIAamp Viral RNA Mini Kit (Qiagen, 52906). Viral titers were quantified using a Quantinova Probe RT-qPCR kit (Qiagen, 208354) with a LightCycler 480 real-time PCR system (Roche) as we previously described [4]. Forward primer for both SARS-CoV-2 and SARS-CoV: 5'-CGCATACAGTCTTRCAGGCT-3'; Reverse primer for both SARS-CoV-2 and SARS-CoV: 5'-GTGTGATGTTGAWATGACATGGTC-3'; SARS-CoV-2-specific probe: 5'-FAM-TTAAGATGTGGTGCTTGCATACGTAGAC-IABkFQ-3'; and SARS-CoV-specific probe: 5'-Cy5-CTTCGTTGCGGTGCCTGTATTAGG-IAbRQSp-3' (Integrated DNA Technologies, Inc) were used. The expression level of ACE2, TMPRSS2, cathepsin L, furin, IFNα, IFNβ, IFNλ1, IL6, TNFα, IP10, and

IL12 in uninfected hNPCs were determined with RT-qPCR with a LightCycler 480 real-time PCR system (Roche). The primer sequences are available upon request.

**Plaque assays**

Supernatants from infected organoids and neurospheres were harvested at 0, 24, 48, and 72 hpi and titrated on Vero E6 cells. After incubation at 37°C for 72 hours, cells were fixed with 10% neutral-buffered formalin. For forming unit (PFU) visualization, fixed samples were stained with 0.5% crystal violet in 25% ethanol/distilled water for 10 minutes for plaque.

**Cytotoxicity assay**

Cell viability was determined by the CellTiter-Glo luminescent cell viability assay (Promega). The kit detects adenosine triphosphate (ATP) levels as a function of cell viability, and was used according to manufacturer's specifications.

**Statistical analysis**

All data were analyzed in GraphPad prism 7.0 software (GraphPad Software Inc., CA, US). Differences between two groups were compared by unpaired Student's *t*-test. Differences among three or more groups were compared by one-way ANOVA. Two-tailed $p$ value < 0.05 was considered statistically significant.

**References:**

1. Chu, H. et al. *ACS Infect Dis* 10.1021/acsinfecdis.9b00526, (2020).
2. Trujillo, C.A. et al. *Cell Stem Cell* **25**, 558-569 (2019).
3. Dang, J. et al. *Cell Stem Cell* **19**, 258-265 (2016).
4. Chu, H. et al. *Lancet Microbe* **1**, e14-e23 (2020).
5. Chu, H. et al. *Clin Infect Dis* 10.1093/cid/ciaa410, (2020).

## Supplementary figures and figure legends:

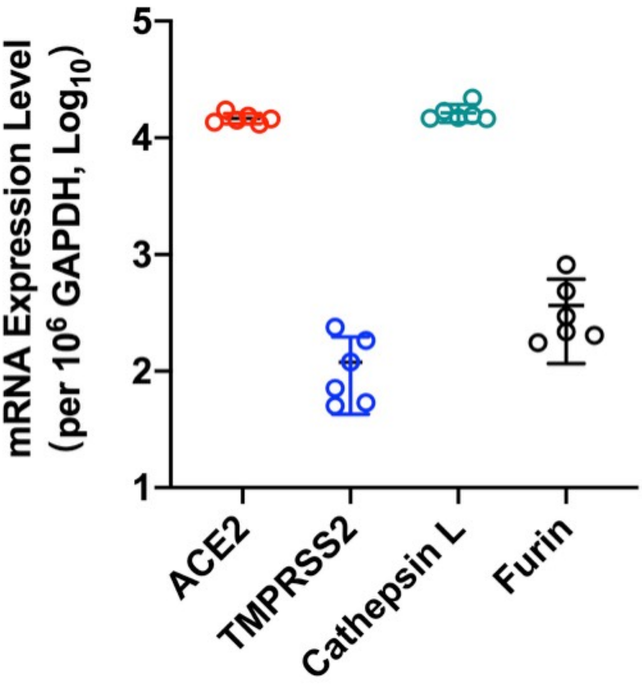


**Figure S1. Expression level of ACE2, TMPRSS2, cathepsin L, and furin in hNPCs.** Bars represent mean ±SD of three independent experiments.

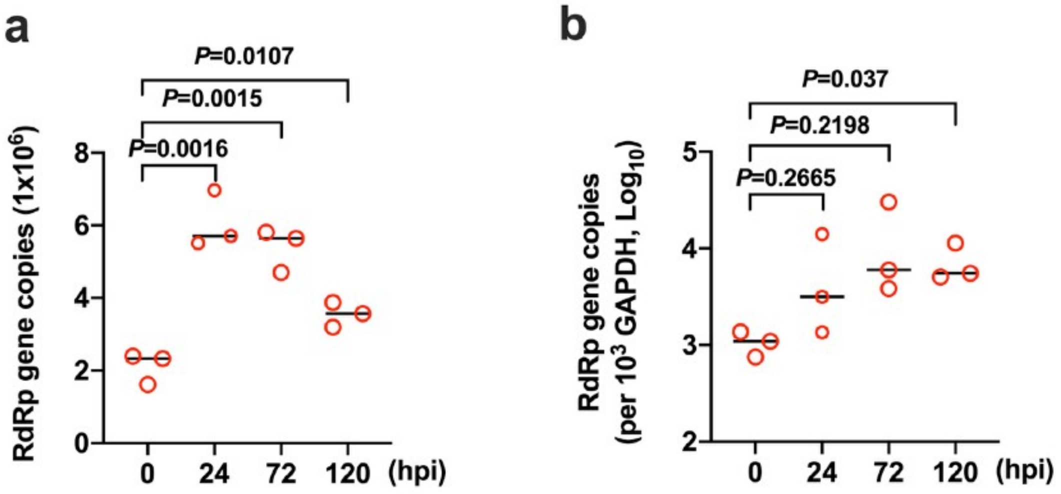


**Fig S2. Replication kinetics of SARS-CoV-2 in hNPCs.**

hNPCs were challenged with SARS-CoV-2 at 10 MOI. Viral supernatant (a) and cell lysate samples (b) were harvested at 0, 24, 72 and 120 hpi and virus gene copies were determined by qRT-PCR. Bars represent mean from three independent experiments.

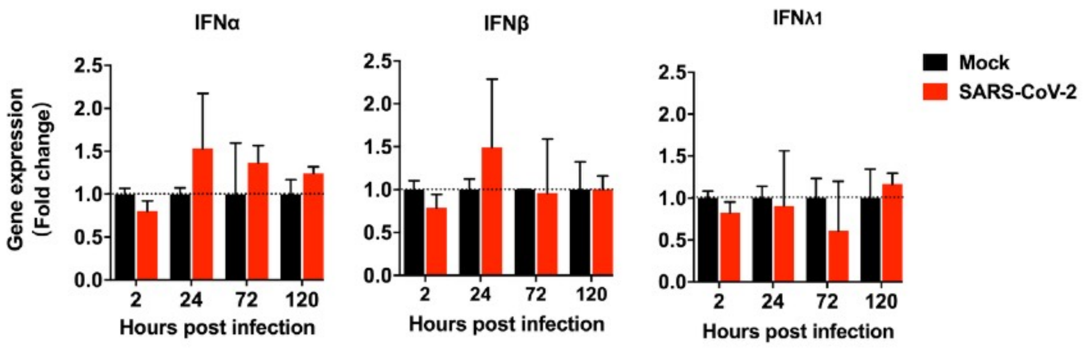


**Figure S3. SARS-CoV-2 did not trigger type I and type III interferon (IFN) response in the infected hNPCs.** hNPCs were challenged with SARS-CoV-2. At 2, 24, and 48 hpi, the infected hNPCs were harvested for qRT-PCR analysis of IFNα, IFNβ and IFNλ1. Bars represented results from three biological replicates from two independent experiments. Statistical significance was determined with two-way ANOVA. SARS-CoV-2 infection did not significantly trigger the expression of any IFN at all evaluated time points.

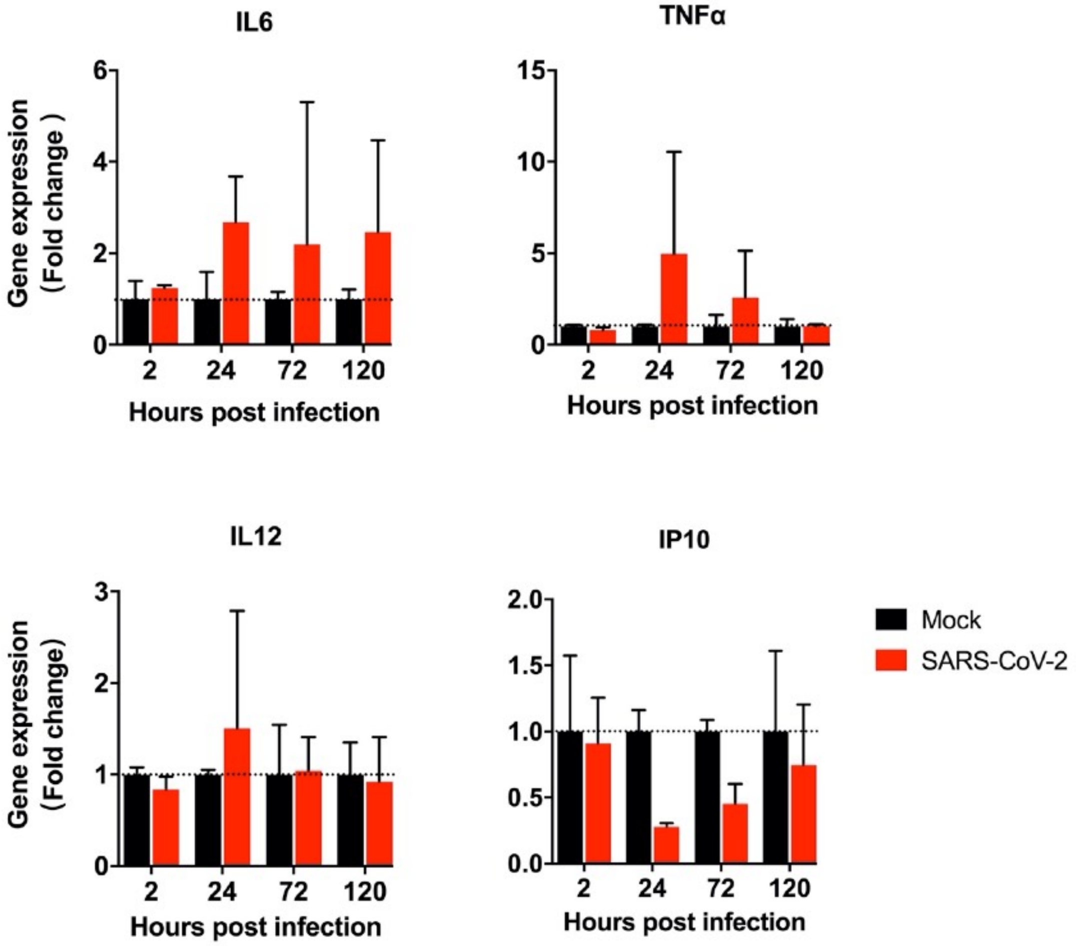


**Figure S4. SARS-CoV-2 did not significantly trigger pro-inflammatory response in the infected hNPCs.** hNPCs were challenged with SARS-CoV-2. At 2, 24, and 48 hpi, the infected hNPCs were harvested for qRT-PCR analysis of representative pro-inflammatory cytokines. Bars represented results from three biological replicates from two independent experiments. Statistical significance was determined with two-way ANOVA. SARS-CoV-2 infection did not significantly trigger the expression of any pro-inflammatory cytokines at all evaluated time points.
